# Editorial

**Published:** 1870-12

**Authors:** 


NEW AGENT FOR THE REMOVAL OF IRON STAINS FROM FABRICS.—A writer in the *Chemical News* says the following method is not attended with the usual bad results, in regard to the destruction of the fibre, which, on account of prolonged contact, ensue when oxalic acid or salt of sorrel are used. The stain must be touched with yellow sulphide of ammonium, by which it will be immediately blackened; after the lapse of a minute or so, wash out the excess of sulphide, and treat the black spot with dilute muriatic acid, by which it is entirely removed; finally, wash well with water.

FISKE FUND PRIZES.—The Trustees of the Fiske Fund offer the following prizes for the year 1871:

First. Ununited Fractures; the conditions under which they occur and the most successful method of treatment.

Second. Hydrate of Chloral; its physiological effects and therapeutical uses.

For the best dissertation on each or either of these subjects the Trustees will pay the sum of one hundred dollars. Essays must be sent to the Secretary of the Fiske Fund Trustees, Dr. S. A. Arnold, Providence, R. I., on or before May 1st, 1871, accompanied by a sealed packet containing the author's name, with corresponding motto or device.

### Mortality for the Month of October, 1870.

| | | | | | |
|---|---|---|---|---|---|
| Accidents, burned by | | " & complications | 2 | Liver, disease of | 1 |
| kerosene | 1 | Diphtheria | 20 | " Induration | 1 |
| " drowned | 4 | Dropsy, general | 5 | " tumor of | 1 |
| " in planing mill | 1 | " (ovarian) | 1 | Lungs, congestion | 4 |
| " fracture of skull | 2 | " chest | 2 | " apoplexy | 1 |
| " thrown from wagon | 1 | Dysentery | 7 | " hæmorrhage | 2 |
| " run over by " | 1 | " chronic | 3 | Meningitis | 6 |
| " railroad | 2 | " typhoid | 1 | " cerebro-spinal | 1 |
| " shot | 1 | Entero-colitis | 1 | Mouth, gan'ous cancer of | 1 |
| Abortion | 1 | Endo-carditis | 1 | Metretis | 1 |
| Apoplexy | 4 | Enteritis | 10 | Old age | 9 |
| Ascites | 1 | " pericarditis | 1 | Paraplegia | 1 |
| Asthma | 1 | " uterus inflammation | 1 | Paralysis | 3 |
| " and disease of heart | 1 | Epilepsy | 1 | Peritonitis | 4 |
| Atelectasis nonatorum | 1 | Exhaustion | 1 | Peritonitis & Colitis | 1 |
| Brain, congestion | 5 | Fever, congestive | 1 | Pneumonia | 7 |
| " inflammation | 5 | " puerperal, | 1 | " typhoid | 2 |
| " softening | 1 | " intermittent | 1 | Pyaemia | 2 |
| Bronchitis | 3 | " scarlet | 11 | Srofula | 2 |
| " capillary | 3 | " " malignant | 2 | Stomatitis | 1 |
| " chronic | 1 | " typhoid | 49 | Suicide by acid | 1 |
| Cancer of liver | 1 | " remittent | 3 | " laudanum | 1 |
| " stomach | 2 | Gangrene of foot | 1 | " morphine | 1 |
| " uterus | 1 | Gastritis | 2 | " severing artery | 1 |
| " kidney | 1 | Gastro-Enteritis | 4 | Syphilis | 1 |
| " Vagina and pancreas | 1 | Heart diseases | 15 | Tabes mesenterica | 28 |
| Chorea and inanition | 1 | Hip-disease | 1 | Teething | 13 |
| Consumption | 53 | Hemiplegia | 1 | Tetanus idiopathic | 1 |
| Convulsions | 52 | Hepatitis, | 1 | Throat inflammation | 1 |
| Croup | 50 | Hydrocephalus | 2 | Uraemia | 1 |
| " diphtheretic | 15 | Inanition | 14 | Ulcer of bladder and ure- | |
| Cholera Infantum | 30 | Intemperance | 4 | thra | 1 |
| Cyanosis | 1 | Intestines gangrene and | | Vitality deficient | 1 |
| Debility general | 6 | strangulation of | 1 | Whooping-cough | 4 |
| Diarrhœa | 17 | Kidneys, Bright's dis- | | " and complications | 1 |
| " chronic | 7 | ease | 1 | Total | 557 |

### COMPARISON.

Deaths in Oct., 1870, 557 | Deaths in Oct., 1869, 601 | Decrease, 44
Deaths in Sept., 1870, 691 | Decrease, 134

### AGES.

| | | | | | |
|---|---|---|---|---|---|
| Under 1 | 153 | 10 to 20 | 26 | 70 to 80 | 12 |
| 1 to 2 | 78 | 20 to 30 | 59 | 80 to 90 | 5 |
| 2 to 3 | 34 | 30 to 40 | 42 | Unknown, | |
| 3 to 4 | 29 | 40 to 50 | 35 | | |
| 4 to 5 | 18 | 50 to 60 | 28 | Total, | 557 |
| 5 to 10 | 24 | 60 to 70 | 14 | | |

Males, 285 | Females, 272 | Total, 557
Single, 406 | Married 151 | Total, 557
White, 552 | Colored, 5 | Total, 557

NATIVITY.

| | | | | | |
|---|---|---|---|---|---|
| Austria | 1 | England | 10 | New Brunswick | 1 |
| Bohemia | 9 | France | 1 | Poland | 1 |
| Canada | 8 | Germany | 74 | Switzerland | 1 |
| Chicago, Native | 65 | Holland | 1 | Scotland, | 10 |
| Chicago, Foreign | 219 | Ireland | 51 | Sweden | 16 |
| U. S., other parts | 73 | Italy | 1 | Unknown | 6 |
| Denmark | 2 | Norway | 6 | Wales | 1 |
| | | | | Total, | 557 |

MORTALITY BY WARDS FOR THE MONTH.

| Wards. | Mortality. | Wards. | Mortality |
|---|---|---|---|
| 1 | 5 | 18 | 41 |
| 2 | 11 | 19 | 14 |
| 3 | 18 | 20 | 22 |
| 4 | 12 | Accidents | 13 |
| 5 | 13 | County Hospital | 13 |
| 6 | 29 | Half Orphan Asylum | 2 |
| 7 | 39 | Hospital Alexian Brothers | |
| 8 | 52 | Immigrants | 2 |
| 9 | 54 | Jewish Hospital | 1 |
| 10 | 15 | Mercy Hospital | 5 |
| 11 | 26 | Police Station | 1 |
| 12 | 20 | Protestant Orphan Asylum | 1 |
| 13 | 5 | St. Joseph Orphan Asylum | 5 |
| 14 | 14 | Suicide | 4 |
| 15 | 64 | | |
| 16 | 27 | Total, | 557 |
| 17 | 29 | | |

---

PULMONARY FETOR.—Dr. Laycock, *Edinburgh Medical Jour.* asserts that there are three distinct kinds of pulmonary fetor, that of ozæna, that of fæces, that of gangrene. The latter, due to putrescent decomposition of pulmonary tissue, is characteristic of true gangrene. The ozæna order is connected with chronic tissue changes of rheumatic origin, as with fibrinous exudation and degeneration. It is found chiefly in fetid bronchitis and bronchorrhœa, and in fetid fibroid vomicæ. The fecal order may also be observed under these circumstances, and has too, probably, a rheumatic origin.—*Richmond and Louisville Medical Journal.*

Receipts from October 25 to November 25, 1870.—Dr. N. Senn, Elmore, Fondulac Co., Wis., 3.00; Dr. A. Hagar, Marengo, Ill., 3.00; Dr. Merriman City, 3.00; Dr. Grosbeck, City, 6.00; Dr. Geo. Fredigke, City, 3.00; Dr. J. F. Young, Dean's Cor., Lake Co., Ill., 3.00; Dr. E. F. Dodge, Fondulac, Wis., 12.00; Dr. C. D. Watson, Covington, Ind., 6.00; Dr. W. W. Duncan, Bible Grove, Ill., 6.00; Dr. H. A. Johnson, City, 10.00; Dr. F. Lemar, Manitowoc, Wis., 9.00; Dr. C. C. Reicharch, Mitchelville, Iowa, 3.00; Dr. Alvis, City, 3.00; Dr. B. Bobb, Dakota, Ill., 6.00.

